# Comparative Effectiveness of Physical and Virtual Reality Simulators in Robotic Surgical Training

**DOI:** 10.3390/jcm15062298

**Published:** 2026-03-17

**Authors:** Gaetano Romano, Fabrizia Calabrò, Carmelina C. Zirafa, Ilaria Ceccarelli, Beatrice Manfredini, Riccardo Morganti, Selene Tognarelli, Francesca Romboni, Arianna Menciassi, Marcello Carlo Ambrogi, Federico Davini, Franca Melfi

**Affiliations:** 1 Division of Thoracic Surgery, Cardiac, Thoracic and Vascular Department, University Hospital of Pisa, 56124 Pisa, Italybeatrice.manfredini91@gmail.com (B.M.); marcello.ambrogi@unipi.it (M.C.A.); 2 Department of Surgical, Clinical, Molecular and Critical Care Pathology, University of Pisa, 56126 Pisa, Italy; fabriziacalabro92@gmail.com (F.C.); i.ceccarelli19@gmail.com (I.C.);; 3 Clinical Trial Statistical Support Unit, University Hospital of Pisa, 56124 Pisa, Italy; 4 The BioRobotics Institute, Scuola Superiore Sant’Anna, 56025 Pisa, Italyarianna.menciassi@santannapisa.it (A.M.); 5 Thoracic Surgery Unit, Cardio-Thoracic-Vascular Department, University Hospital “SS. Annunziata”, 87100 Cosenza, Italy

**Keywords:** robotic surgery, thoracic surgery, training, simulation, physical simulation, technology, surgery residency

## Abstract

**Introduction**: Robotic surgery training requires effective simulation methods to ensure proficiency. Virtual reality (VR) simulators and physical models offer different approaches. **Methods**: A study was conducted with 30 surgical residents, divided into two groups: one trained on a high-fidelity physical simulator and the other on a VR simulator. Both groups completed standardized exercises, followed by an assessment of their surgical performance using the da Vinci Surgical System simulator. Performance scores were analyzed using statistical methods, including *t*-tests and multiple linear regression. **Results**: Residents trained on the physical simulator obtained higher scores compared with those using VR simulation, with a statistically significant difference in overall scores (76 ± 17 vs. 34 ± 29; *p* < 0.001). The use of the physical simulator was the most influential factor in improved performance, independent of the year of residency. **Conclusions**: High-fidelity physical simulators enhance robotic surgical training compared to VR simulators.

## 1. Introduction

### 1.1. Simulation in Robotic Surgery

At present, several tools are available for simulation in robotic surgery, including virtual simulators, animals and synthetic models.

The main virtual simulators for robotic surgery are:

- dVSS, da Vinci Skills Simulator (Intuitive Surgical Inc., Sunnyvale, CA)
- dV-Trainer (Mimic Technologies, Inc., Seattle, WA)
- RoSS, Robotic Surgical Simulator (Simulated Surgical Systems, LLC, Williamsville, NY)
- RobotiX Mentor (Simbionix USA Inc., Cleveland, OH)

These four simulators, which are currently available on the market, simulate the da Vinci^®^ robot [1], allowing surgeons in training to be able to exercise without creating risks for patients. Furthermore, the exercises can be repeated several times, going from the simplest to the most complex. Additionally, it is possible to evaluate the surgeon’s performance and trace their learning curve over time.

Practicing on a virtual simulator also allows the surgeon to learn basic skills such as dissecting tissue, cutting or suturing.

Some exercises focus on these simple tasks, which are the foundations of surgical operations. Other exercises, which are procedure-based, are more complex and can simulate an entire surgery or one of its steps, combining different skills [2].

Furthermore, it has been hypothesized that performance in the virtual simulation could predict performance in the operating room. However, this aspect requires further investigation, given that it is not easy to assess since surgical performance depends on many variables, including the characteristics of the patient and the decision-making abilities of the operator [3].

Da Vinci Skills Simulator, produced by Intuitive Surgical, physically looks like a “suitcase”, which is docked externally to the da Vinci^®^ surgical console and connected to it via a fiber optic cable.

Thanks to its design, the console can be easily converted into a simulator, with the dVSS replacing the vision cart and the patient cart.

To date, versions compatible with both da Vinci Si and da Vinci Xi consoles have been developed, providing similar exercises. Each version is specific to the single model, so they are not interchangeable.

The exercises were developed in collaboration with Mimic Technologies and Simbionix. For the Da Vinci virtual simulator, 41 exercises are available. The exercises are divided into categories with different levels of difficulty: endowrist manipulation 1, camera and clutching, endowrist manipulation 2, energy and dissection, needle control, needle driving, games and suturing skills.

For the Xi model, 47 exercises divided into 8 categories are available.

Each of the simulation systems is provided with explanatory videos prior to all exercises.

With either tool, the score obtained is revealed at the end of each exercise.

The Xi virtual simulator has better graphics and more features than the Si model; for example, it allows administrators to create customized routes for surgeons in training.

Furthermore, the administrator, through a special program, can create different user profiles and download performance data for analysis [1].

### 1.2. Real Models

Simulations can be carried out on real models, such as human cadavers and living animals. To date, the availability of human cadavers in certain countries is very limited, given the small number of donations to scientific research.

Therefore, the high costs and logistical difficulties that derive from finding cadavers mean that they are not an adequate and widely available tool for surgical simulations.

Simulation on live animals, on the other hand, raises ethical questions, which together with anatomical differences represent an important limitation [4].

At present, thanks to new technologies, including 3D printers, it has become possible to develop synthetic models, which limit the use of animals in surgical simulations [5].

An example is the chest model developed by Touchstone 3D in collaboration with KindHeart. In this model, animal tissues can be positioned to simulate several procedures, such as lymph node dissection or lobectomy.

In 2017, Francisco Schlottmann and Marco G. Patti published one of the first studies concerning the use of a robotic system on a real model [4].

In this study, the authors used a perfused animal tissue block, which was modified to increase its similarity to human anatomy and integrated in a bench model which simulates a real human body.

The block included: heart, lungs, aorta, esophagus, stomach, duodenum, diaphragm, liver and spleen. The aorta was cannulated and perfused with artificial blood. This type of real model allows the surgeon to not only to learn the basic skills necessary for any type of robotic operation, but also to replicate an entire surgical procedure starting from the docking of the robotic system.

In the mentioned study, 10 trainees performed a Nissen fundoplication under the supervision of an expert surgeon. The results have been positive; in fact, it was considered a realistic simulator and an excellent training tool by the study participants [4].

Subsequently, Schlottmann and Patti with Jason M. Long and Sean Brown assessed the confidence level of 20 residents before and after using the simulator through a questionnaire.

In this study, trainees had to follow a three-day course in which they would perform different procedures on the tissue block integrated in the model, such as Nissen fundoplication, Heller myotomy, partial vertical gastrectomy, colectomy and lobectomy. The study demonstrated that the confidence level after simulation was increased.

This study consequently suggests the introduction of simulations on real models in training courses for robotic surgery, in addition to VR simulators, to increase surgeons’ confidence with the robotic system [6].

In 2017, another paper in which the model was completely synthetic and made of polyvinyl alcohol was published. This study aimed to create a synthetic heart with a human-like structure and consistency, to enable simulation and training in the context of minimally invasive cardiac surgery [7].

Robotic surgery differs considerably from other surgical approaches and, consequently, requires adequate proficiency. In the late 1990s, the Society of American Gastrointestinal Endoscopic Surgery (SAGES) set up a committee to determine the Fundamentals of Laparoscopic Surgery (FLS) [8]. In 2004, the FLS curriculum was promoted by SAGES and by the ACS (American College of Surgeons) as a minimum standard for surgeons who want to perform laparoscopic surgery. In the United States, a certification with positive evaluation for the FLS curriculum is even required to perform the certification exam in general surgery residency [9].

For these reasons, it is necessary to validate an adequate training path for robotic surgery in all specialties.

In a robotic training course, surgeons must be provided with theoretical elements on the functioning of the robotic system, starting from docking through to realizing the potential of the robot. Moreover, the learner must be able to develop the skills for the management of an adverse event.

For the practical component of the training course, the initial step is represented by simulations, first in virtual reality and then on dry-lab and wet-lab models.

Finally, surgical trainees should be guaranteed the opportunity to perform real procedures, beginning with simple individual steps and progressively advancing to more complex phases, ultimately performing the entire operation under the supervision of an experienced surgeon. The degree of difficulty must also gradually increase, with reference to the anatomical characteristics of the patient and the type of procedure performed [10].

At present, several curricula which are specific for robotic surgery have been validated, including a training program offered by Intuitive Surgical.

The training pathway is an online course divided into four parts: the first phase consists of an introduction to the robotic system, while the second involves the development of technical skills through online courses, simulations and practical training. The third and fourth phases foresee a progressive advancement of clinical skills: the surgeon in training must begin to integrate the robotic system into their clinical practice under the supervision of official proctors, progressively increasing their skills and competences.

In 2020, the results of a validation study conducted by the Institute for Surgical Excellence (ISE) were published with the participation of the major ACS-accredited education centers.

Among those who had followed the FSR program, better results were obtained in terms of performance compared to control cases, thus demonstrating the effectiveness of the FSR curriculum [11].

Several consensus conferences have led to the creation of the FRS curriculum which, as mentioned above, is currently considered an effective and valid path for the acquisition of basic robotic skills.

However, more specific curricula are required for the different surgical specialties, which is why the ISE has started working on the creation of the Fundamentals of Robotic Gynecologic Surgery (FRGS) curriculum and the Fundamentals of Thoracic Robotic Surgery (FTRS).

To date, there is still no standardized curriculum for robotic thoracic surgery, but the ISE has induced the formation of a committee for the creation of the FTRS curriculum, starting from what has already been achieved for the FRS.

In light of the aforementioned considerations, we deemed it necessary to conduct a study aimed at standardizing a training program for robotic surgery, focusing on young surgical residents who are specifically involved in the use of the robotic system.

For this purpose, we used the dVSS (virtual simulator for da Vinci robotic system) and a prototype, created in collaboration with the Bio-Robotic Institute of Sant’Anna School of Advanced Studies of Pisa, Italy. Both simulators, the virtual and the physical model, are available at the Multidisciplinary Center for Robotic Surgery in the University Hospital of Pisa, Italy.

**Figure 1:**
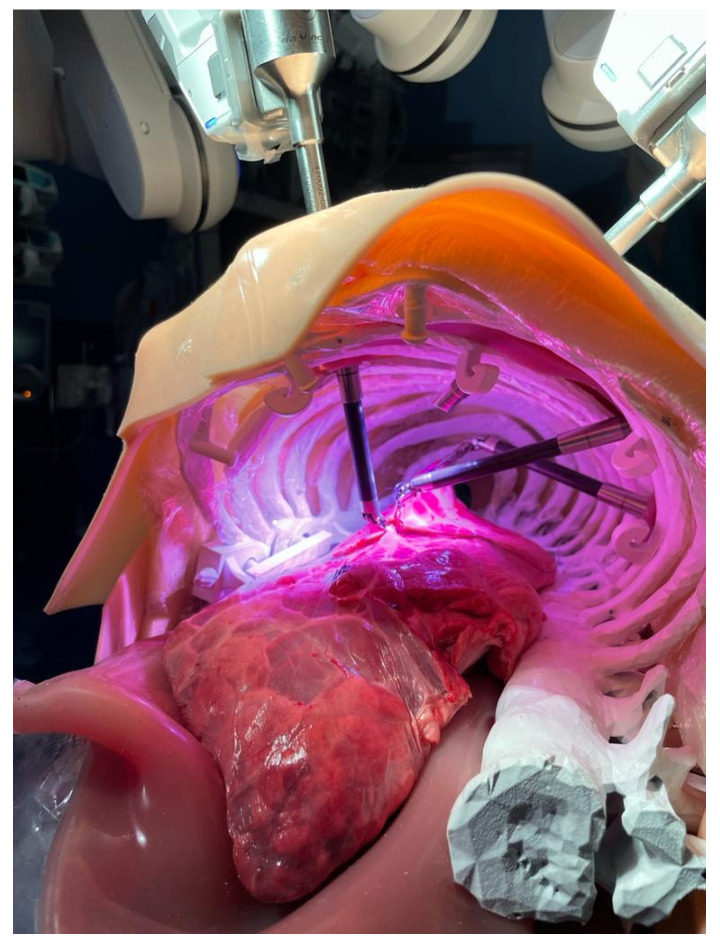
Robotic set up for the physical model.

The model, shown in Figure 1, reproduces the thorax of an adult man of average build with high fidelity, in which animal lungs can be integrated.

## 2. Materials and Methods

The primary objective of this study was to evaluate whether training with a high-fidelity physical simulator results in robotic console performance compared with standard virtual simulation training alone.

Secondary objectives included assessing the influence of residency year and baseline technical performance on the final overall score.

We hypothesized that residents trained using the physical simulation model would achieve significantly higher performance scores in the final standardized robotic task, independently of baseline skill level and year of residency.

Between June 2021 and September 2022, a total of 30 surgical residents in their first three years of training from various surgical disciplines were voluntarily enrolled in the study. The participants (18 males and 12 females) were distributed as follows: thoracic surgery (n = 8), urology (n = 2), and general surgery (n = 19).

Based on their year of residency, the trainees were categorized as follows: 12 had completed their first year, 8 their second, and 10 their third year. Subsequently, the participants were assigned to two groups (Group 1 and Group 2). All residents were naïve to robotic surgery as first operators, and were appropriately randomized into the two study groups after stratification by year of residency. All participants provided written informed consent prior to enrollment in the study.

### 2.1. Study Protocol

The study was structured into three distinct phases.

- Phase I: All participants completed a set of standardized exercises on a virtual simulator. These exercises were selected in collaboration with robotic system engineers to assess core competencies in robotic console usage.
- Phase II: The participants were divided into two groups, ensuring an equitable distribution based on residency year. Group 1 trained on a physical simulator, while Group 2 trained on a virtual simulator.
- Phase III: All residents performed the same exercise on the virtual simulator to compare the outcomes between the two groups.

#### 2.1.1. Phase I

During the first phase, which was identical for both groups, three exercises were performed using the da Vinci Surgical System (dVSS):

1. Camera and Clutching—Ring Walk 1
2. Energy and Dissection—Energy Switching 1
3. Suturing Skills—Single Vertical Suture

Table 1 outlines the parameters evaluated for each exercise.

- The Ring Walk 1 exercise required participants to center a sphere on a target while maneuvering the camera and moving a ring along a guided pathway to a specified target.
- In the Energy Switching 1 exercise, participants had to identify and target small blue and yellow spheres inside a virtual cavity by adjusting the camera. Once identified, they applied monopolar or bipolar energy, depending on the sphere’s color, until the target disappeared.
- The Single Vertical Suture exercise required the execution of two square knot sutures on a virtual pad with a vertical defect.

**Table 1:** Evaluated skills in phase One.

Ring Walk 1 (Camera and Clutching)	Time to complete (s)
	Economy of motion (cm)
	Instrument collisions
	Excessive instrument force (s)
	Instrument out of view (cm)
	Master workspace range (cm)
	Overall score (%)
Energy Switching 1 (Energy and Dissection)	Time to complete (s)
	Economy of motion (cm)
	Instrument collisions
	Excessive instrument force (s)
	Instrument out of view (cm)
	Master workspace range (cm)
	Misapplied energy time (s)
	Overall score (%)
Single Vertical Suture (Suturing Skills)	Knot tying
	Needle handling
	Time efficiency
	Time to complete (s)

#### 2.1.2. Phase II

**Figure 2:**
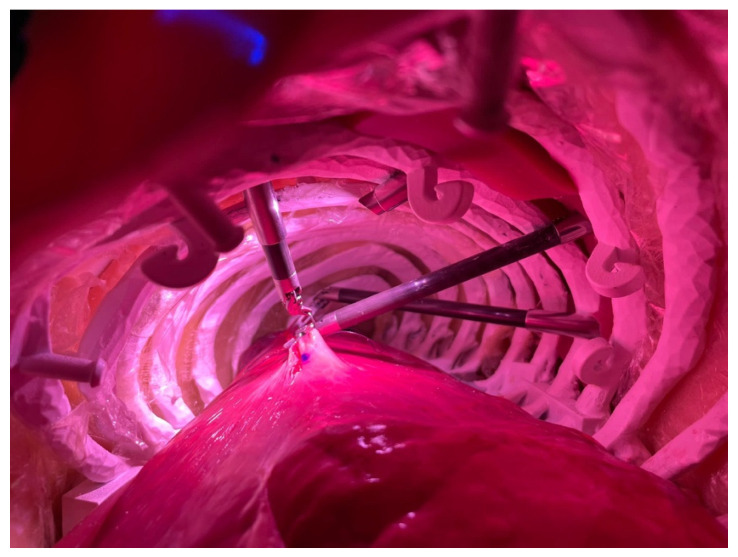
Robotic continuous suture using physical model.

The second phase differed between the two groups:

- Group 1 performed a continuous suture in the operating room using the fourth-generation robotic system on an ex vivo animal lung integrated within a physical chest model as shown in Figure 1 and Figure 2.
- Group 2 performed the Continuous Suturing exercise on the dVSS, which serves as a virtual analog of the task performed by Group 1.

Table 2 presents the parameters evaluated in this phase. Group 1 practiced on the da Vinci Xi robotic system in an operative setting, using a chest model integrated with bovine lungs to simulate human lung tissue. The parameters assessed included suture execution time, completion status, and the number of suture threads required for complete defect repair.

Participants in Group 1 were required to perform docking procedures before transitioning to the surgical console to execute a continuous suture along a 5 cm incision on the animal lung. The task also involved controlling the fourth robotic arm to correctly position the lung parenchyma, ensuring optimal exposure of the defect.

Conversely, Group 2 performed the Continuous Suturing exercise on the dVSS, simulating a continuous suture on a vertically positioned virtual pad.

#### 2.1.3. Phase III

The final phase was conducted using the dVSS simulator for both groups. The selected exercise, Three Arm Relay 1, assessed the ability to perform a high-complexity task requiring integration of multiple functions, including camera movement, clutching, pedal control, and use of the fourth arm.

Performance was assessed using the dVSS platform, which provides standardized and objective automated metrics. Although no independent external rating system was employed, the use of a single validated simulator ensured reproducibility and minimized observer-related variability.

For this study, our institution does not require approval from the Ethics Committee, as it does not involve patients or the use of sensitive data. Bovine lungs used for the simulation exercises were obtained from the standard food supply chain; no animals were sacrificed for research purposes. All training activities were conducted in dedicated simulation facilities using equipment reserved exclusively for educational use.

### 2.2. Scoring System

At the end of each exercise, the dVSS simulator generated an individual performance score. Several parameters were evaluated across different exercises, including completion time, motion efficiency, and excessive force application. Additional parameters were specific to each exercise type.

The dVSS simulator reports scores using the Classic System, which expresses the overall score as a percentage of the maximum attainable score.

### 2.3. Statistical Analysis

Categorical variables are summarized using absolute frequencies and percentages, while continuous variables are expressed as mean ± standard deviation.

To assess the relationship between the “Physical Simulator” variable (No vs. Yes) and the overall score, an independent two-tailed *t*-test was performed. Pearson’s correlation analysis was conducted to examine the association between “Year of Residency” (1st–3rd year) and the overall score. A multiple linear regression model was then applied to analyze the combined effect of these independent variables on the overall score.

A significance level of *p* < 0.05 was set for all analyses, which were conducted using SPSS v.28 software.

## 3. Results

The scores obtained by the residents in phase three task “Three arm relay 1” were evaluated in terms of overall score expressed as a percentage.

Group 1, consisting of residents who performed phase two using the physical simulator, obtained an average of 76 (SD ± 17), while group 2 achieved an average score of 34 (SD ± 29).

The univariate analysis using the *t*-test for independent samples showed a significant *p*-value (<0.001).

We additionally performed a multivariate analysis including the three baseline exercises as independent variables and the overall score obtained in Phase Three as the dependent outcome.

This analysis demonstrated that the mean scores achieved during Phase One did not significantly influence the overall score in Phase Three.

These results are summarized in Table 3.

Moreover, Pearson correlation analysis was performed in order to compare the year of residency with the Overall score. A Pearson r value of 0.358 was obtained, with a non-significant *p*-value (*p*-value = 0.052).

It is important to note that Pearson’s correlation reflects the unadjusted association between variables. In contrast, the multivariate regression model estimates the independent effect of residency year after controlling for the use of the physical simulator. Adjustment for concomitant variables may reduce unexplained variance and clarify the specific contribution of each predictor, thereby modifying statistical significance.

Subsequently, a multiple linear regression was performed, relating the two independent variables: “Physical simulator” and “Year of residency” with the Overall score.

From this analysis, a regression coefficient of 36.177 with a significant *p*-value (*p*-value 0.001) for the “Physical simulator” variable was obtained. In addition, concerning the “Year of residency” variable, a regression coefficient of 12.171 with a *p*-value of 0.024 was achieved.

**Figure 3:**
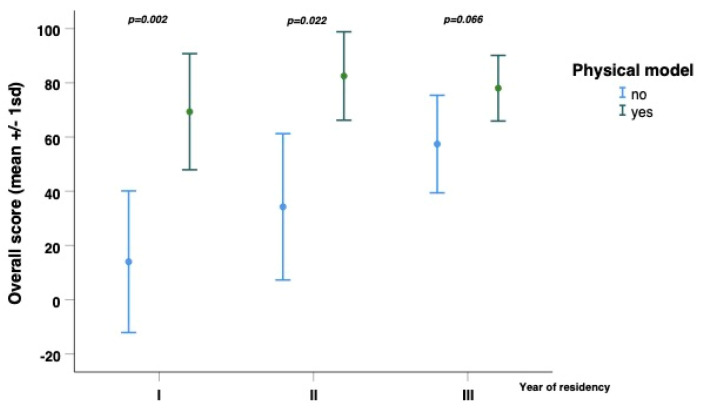
Comparison between Simulator and Overall score stratified by year of residency.

The results are summarized in Figure 3 and Table 3.

## 4. Discussion

Robotic surgery represents the latest frontier in minimally invasive surgical techniques, offering enhanced precision, improved dexterity, and better ergonomics for surgeons. As robotic-assisted procedures continue to expand across various surgical specialties, the need for a structured and comprehensive training pathway for young surgeons has become imperative. Among the available training methods, virtual reality (VR) simulators have been widely adopted to facilitate the acquisition of fundamental psychomotor skills in a controlled and risk-free environment. These simulators provide an essential first step in the learning curve, allowing trainees to develop hand–eye coordination and instrument handling techniques without posing any threat to patient safety.

However, despite their advantages, VR simulators remain limited in their ability to fully replicate the real-world experience of operating with an actual robotic system on live tissues. The haptic feedback, depth perception and tissue interaction encountered in a real surgical setting are difficult to emulate in a purely virtual environment. As an alternative, training on real models, including human cadavers and live animals, has been explored. Nevertheless, these methods present significant ethical, logistical, and financial constraints, making them less feasible as routine training tools. In contrast, synthetic models have emerged as a more sustainable and ethically sound option for robotic surgery training, offering a compromise between realism and accessibility.

A notable study conducted by Raison et al. in 2020 compared the efficacy of VR simulation (dVSS) with dry-lab simulation using the da Vinci Xi robotic system [11]. Their study was structured into three phases: the first phase focused on acquiring fundamental robotic surgical skills, while the second phase involved performing a suture. In the third phase, both groups executed a ureterovesical anastomosis on a synthetic model. Performance was evaluated using the Global Evaluative Assessment of Robotic Skills (GEARS) system. The findings demonstrated that both training modalities resulted in performance improvements, as evidenced by increased GEARS scores. However, a significant advantage was observed in favor of dry-lab simulation, particularly in the third phase, aligning with the outcomes of our present study.

Similarly, a 2014 study by Amirian et al. compared VR-based training with dry-lab simulation. In the initial phase, participants were evaluated on their ability to perform a suture using a robotic system [12]. They were then divided into two groups, one trained on a VR simulator and the other on a dry-lab model. In the final phase, all participants were required to perform a robotic suture again. The results indicated improvements in both groups, with a slight advantage for the dry-lab training; however, the difference did not reach statistical significance. These findings partially corroborate the conclusions drawn from our study.

The present study aimed to validate the use of a physical simulator in the robotic surgical training of young surgeons, with the broader objective of establishing a standardized and effective training program. The analysis of performance scores in the third phase of our protocol revealed a statistically significant advantage in favor of the group trained on the real model. Specifically, participants in Group 1, who underwent training with the physical simulator during the second phase, achieved higher overall scores compared to Group 2. This finding underscores the critical role of physical simulation in enhancing robotic surgical proficiency.

Furthermore, multivariate analysis demonstrated that exposure to a physical simulator significantly influenced surgical expertise, particularly in relation to the year of residency.

Moreover, we conducted a multivariate analysis incorporating the results of the three baseline exercises as covariates, in order to assess their potential influence on the overall score achieved in Phase Three. This analysis clearly demonstrated that baseline performance did not exert any statistically significant effect on the final overall score.

This finding is particularly meaningful, as it strengthens the internal validity of our study. Specifically, it confirms that the differences observed in overall performance cannot be attributed to pre-existing disparities in technical skills at baseline. By controlling for initial performance levels, we were able to more accurately isolate the true determinants of improvement.

Importantly, the data indicate that the use of the physical model represents an independent predictor of enhanced performance outcomes. The persistence of its statistical significance after adjustment for baseline ability supports the hypothesis that structured simulation-based training provides an added educational benefit beyond inherent or previously acquired skills. In parallel, the significant association between year of specialization and performance is consistent with the expected progressive acquisition of technical competence through cumulative clinical exposure and structured surgical training.

Taken together, these findings suggest that the integration of a physical simulation model into surgical training programs may provide a measurable and independent advantage in skill acquisition. From an educational and clinical perspective, this supports the implementation of simulation-based strategies as a complementary tool within residency curricula, with the potential to enhance procedural proficiency in a structured, reproducible, and competency-oriented framework.

However, an important methodological consideration is the partial heterogeneity of elemental skills across the training exercises. The virtual suturing module primarily focuses on needle driving within a confined operative field, with limited requirement for extensive camera repositioning, clutching, or systematic fourth-arm use. In contrast, the physical simulator allows greater spatial freedom and more dynamic multi-arm coordination.

The final task required integrated management of the robotic system, including retraction arm use, camera control, clutching, and collision avoidance. Although the physical simulator may reproduce several of these elements more closely, the final assessment was conducted on the virtual platform, potentially favoring participants with greater familiarity with that interface.

Overall, while these differences should be acknowledged, the protocol was intentionally designed to evaluate skill transfer across environments with varying technical demands. Therefore, the observed performance differences are likely attributable to the added training value of a more spatially complex simulation setting, rather than to a systematic task imbalance.

This finding may reflect a positive transfer of training, whereby exposure to a more physically realistic and spatially demanding environment enhances integrated robotic skills that generalize across simulation modalities.

In addition, although the participants were recruited from different surgical specialties, all were naïve to robotic surgery as first operators, and robotic console skills are platform-specific rather than procedure-dependent. Moreover, baseline performance did not influence the final outcome, suggesting that specialty background did not act as a significant confounder.

These observations highlight the pivotal role of structured training pathways in shaping the learning curve of robotic surgery. By incorporating physical simulation into residency programs, surgical trainees may experience accelerated skill acquisition and improved clinical performance, ultimately leading to better patient outcomes.

Future research should focus on optimizing the integration of different training modalities—VR, dry-lab, and physical simulation—to develop a comprehensive and scalable curriculum for robotic surgery education. Additionally, long-term studies assessing the impacts of various training methods on clinical performance in real surgical settings would provide valuable insights into the most effective strategies for surgical skill development.

The results of this work demonstrate that a high-fidelity physical simulator, which reproduces the human chest, can be considered a valid instrument in the specific training path for robotic surgery, with a statistically significant difference when compared to the virtual reality simulator.

The standardization of the training course with the related results will be the focus of future studies.

## 5. Limitations

This study has several limitations that should be acknowledged. First, the sample size was limited to residents available within a single academic training center, and no a priori power calculation was performed, which may affect the generalizability of the findings. Second, performance assessment was conducted exclusively using the dVSS platform, without external expert rating or evaluation in a real operative clinical setting. Although this ensured objective and standardized scoring, it does not allow for direct extrapolation to actual surgical performance. Third, the participants were recruited from different surgical specialties; however, all were naïve to robotic surgery as first operators, and baseline performance was controlled for in the multivariate analysis. Finally, the study evaluated short-term skill acquisition, and long-term retention of technical proficiency was not assessed. Future multicenter studies with larger cohorts and longitudinal follow-up would be valuable to confirm and extend these findings.

## Figures and Tables

**Table 2:** Evaluated parameters in Phase two.

Suture on Real Lung Group 1	Time of surgery
	Number of damaged needles
	Suture completed (yes/no)
Continuous Suturing Group 2	Total time (s)
	Efficiency subtotal
	Total number of needle passages
	Number of unnecessary needle piercing points
	Number of times the grasped needle was taken outside the visible field
	Number of dropped needles
	Suture break count
	Suture tail grabs count
	Reset count
	Knot granny count
	Penalty subtotal
	Overall score

**Table 3:** Univariate and multivariate analysis of the “Overall Score” influencing factors (range: 0–100).

Factor	Mean (SD) or Pearson, s r	Univariate *p*-Value	CR	95% CI	Multivariate *p*-Value
Physical simulator		<0.001	36.18	17; 55	0.001
(0) no	34 (29)				
(1) yes	76 (17)				
Year (range: 1–3)	0.358	0.052	12.17	1.7; 22.6	0.024
First exercise, Phase I (range: 0–100)	0.254	0.151	0.13	−0.35; 0.61	0.581
Second exercise, Phase I (range: 0–100)	0.42	0.021	0.257	−0.61; 1.12	0.545
Third exercise, Phase I (min)	−0.374	0.042	−0.586	−3.39; 2.22	0.670
Constant of the linear model			−4.54	−853; 76	0.908

## Data Availability

All data are available upon request directly to the authors.
